# Pseudoangioedema as a presenting symptom of dermatomyositis with antinuclear matrix protein 2 autoantibodies

**DOI:** 10.1016/j.jdcr.2024.12.018

**Published:** 2025-01-29

**Authors:** Han Li, Steven Svoboda, Kiran Motaparthi

**Affiliations:** a Department of Medicine, Hospital Medicine, Wellstar Kennestone Regional Medical Center, Marietta, Georgia; b Department of Dermatology, University of Florida College of Medicine, Gainesville, Florida

**Keywords:** angioedema, autoantibodies, dermatomyositis, idiopathic inflammatory myopathy, juvenile dermatomyositis, myopathy, pseudoangioedema

## Introduction

Dermatomyositis (DM) is an inflammatory myopathy with characteristic cutaneous findings of heliotrope rash, Gottron papules and sign, periungual erythema, calcinosis cutis, telangiectasias, and ulcers. DM is associated with several autoantibodies, such as antinuclear matrix protein 2 (NXP-2), antitranscriptional intermediary factor 1, and antimelanoma differentiation-associated protein 5; cutaneous manifestations vary depending upon the associated autoantibodies.^1^ NXP-2 DM is associated with a higher prevalence of severe myositis, malignancy in adults, and atypical cutaneous findings, which, when present, include calcinosis and subcutaneous edema.^2^ We present a case of NXP-2 DM presenting with pseudoangioedema of the lips.

## Case presentation

**Fig 1:**
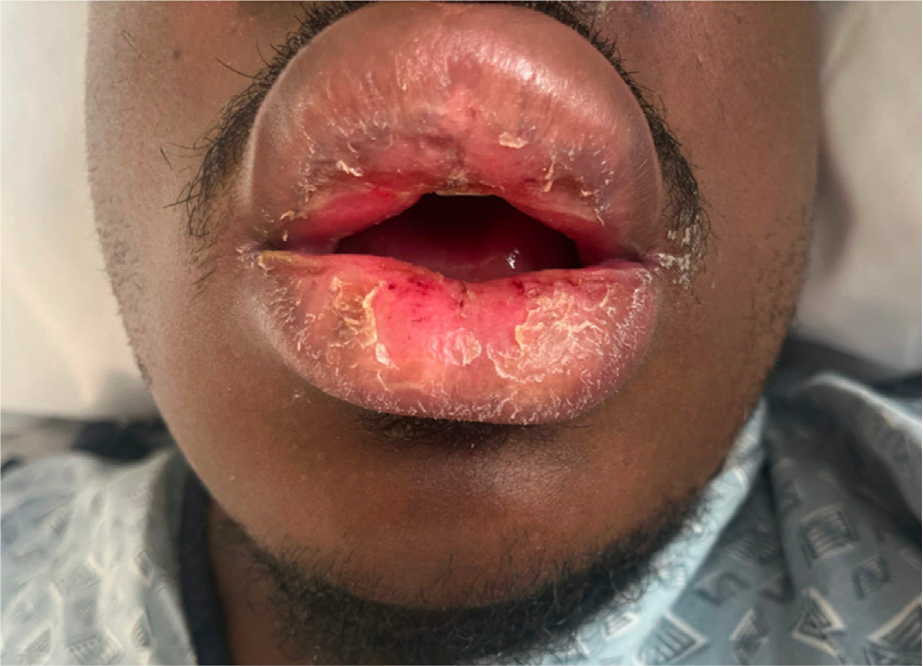
Marked edema (pseudoangioedema) of the lips.

**Fig 2:**
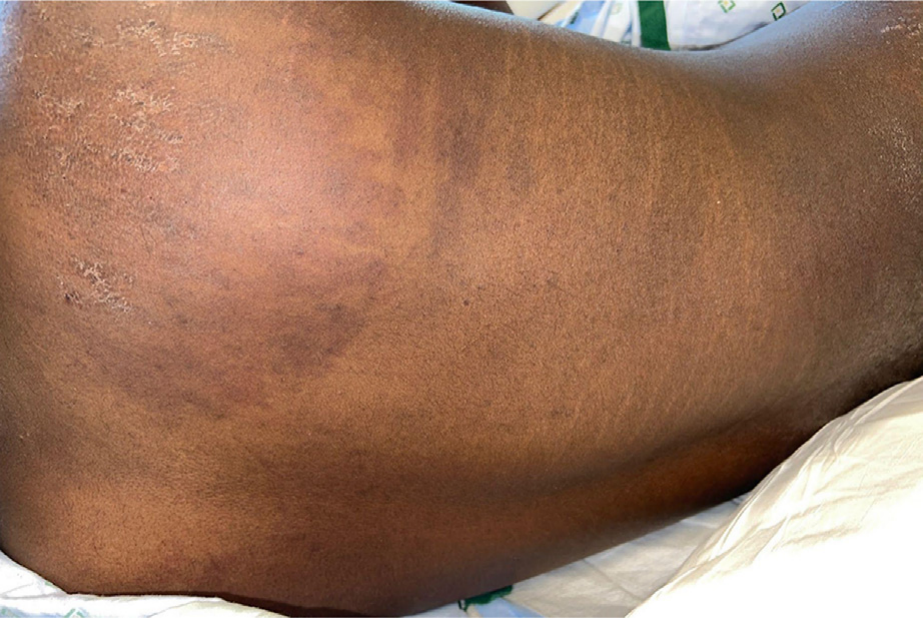
Flagellate erythema on the upper back.

**Fig 3:**
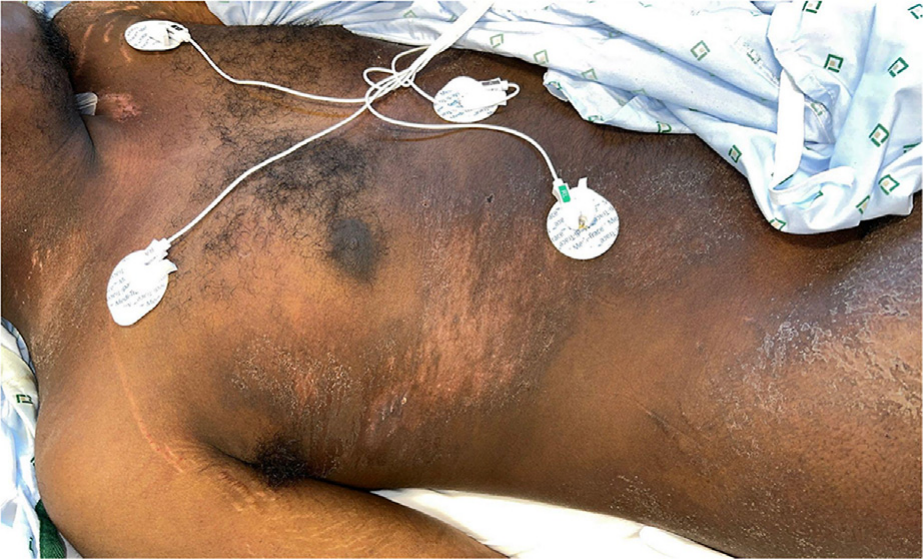
Poikilodermatous to hyperpigmented plaques over the chest, abdomen, flank, and thigh.

**Fig 4:**
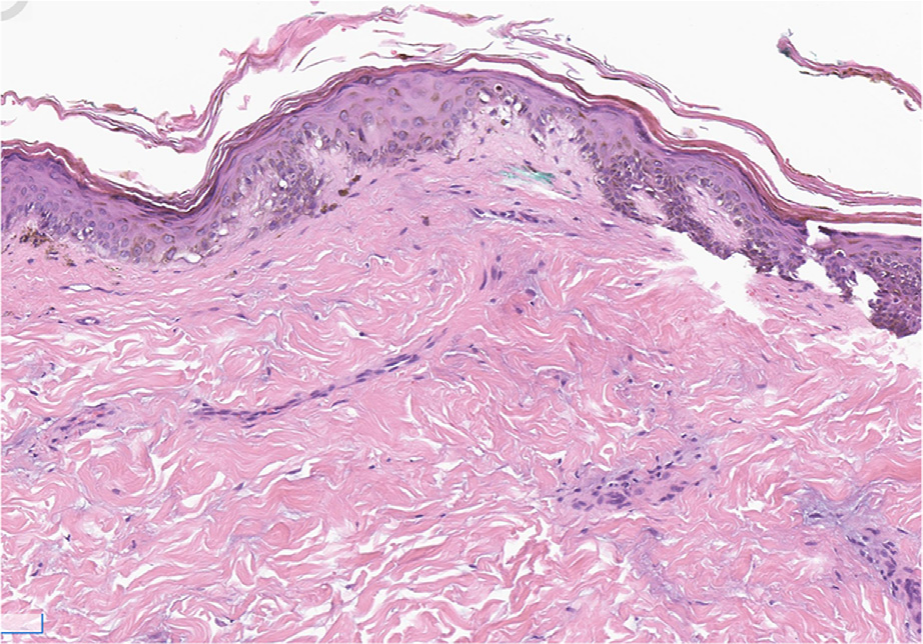
Cell poor vacuolar interface dermatitis with increased dermal mucin and perivascular lymphocytic infiltrate (H&E, 200× magnification).

A 21-year-old man with no significant medical history presented with 2 months of progressive proximal muscle weakness in upper and lower extremities, lip swelling, dysphagia, and a rash on the upper arms, thighs, neck, chest, and back. The patient denied taking any medications in the months preceding symptom onset. Physical examination was notable for marked edema of the lips (Fig 1) and poikilodermatous plaques on the back, flanks, thighs, and neck and shoulders (Figs 2 and 3). No heliotrope rash, Gottron papules, or nail fold changes were observed. Laboratory findings were significant for a creatine kinase of 3458 units/liter and lactate dehydrogenase of 605 units/liter. Electromyography was consistent with a myopathic process. Histopathology demonstrated a cell-poor vacuolar interface dermatitis with increased dermal mucin and perivascular lymphocytic infiltrate (Fig 4). A myositis antibody panel demonstrated high positivity for anti-NXP-2 antibodies. Computed tomography of the chest and abdomen did not reveal evidence of interstitial lung disease or malignancy, and ultrasound of the scrotum was unremarkable. Computed tomography of the neck showed diffuse intramuscular edema of the prevertebral musculature. Intravenous immunoglobulin was initiated at a dose of 2 g/kg intravenous divided over 4 days and pulse methylprednisolone 1 g intravenous daily for 5 days followed by prednisone at 1 mg/kg with marked improvement in symptoms including edema and dysphagia.

## Discussion

The features of DM vary depending on the underlying serologic subtype. DM associated with anti-NXP-2 antibodies has a bimodal age distribution, with a 20% to 25% prevalence in juvenile dermatomyositis and a 2% to 25% prevalence in adult DM.^3^ While anti-NXP-2 DM in older adults is associated with classic features including Gottron papules and heliotrope rash, the juvenile variant is marked by disabling myopathy, calcinosis cutis, and periorbital edema. As an isolated finding, the differential diagnosis of pseudoangioedema of the lips would include granulomatous cheilitis (GC) and disorders associated with GC, such as Melkersson-Rosenthal syndrome, sarcoidosis, and Crohn disease. However, the presenting symptoms and remainder of physical examination findings were characteristic of DM but could not be explained by GC or disorders associated with GC. Anti-NXP-2 DM is strongly associated with malignancy in older adults, with cancer prevalence increasing in an age-dependent manner from below 25% in ages 20-30 up to 55% in ages over 60.^4^ Interstitial lung disease is relatively rare in anti-NXP-2 DM compared to other DM subtypes.^5^ The mainstay of treatment comprises glucocorticoids and intravenous immunoglobulin for severe myopathy, with methotrexate and rituximab serving as adjunct therapies or steroid-sparing agents.

Periorbital edema, dysphagia, and generalized subcutaneous edema have previously been described in association with anti-NXP-2 DM.^2^^,^^5^^,^^6^ The mechanism of subcutaneous edema is unknown but is postulated to reflect increased subcutaneous vascular permeability secondary to immune complex deposition and elevated vascular endothelial growth factor expression.^7^ Pseudoangioedema has been described in association with antimelanoma differentiation-associated protein 5, anti-SAE, and antitranscriptional intermediary factor 1 antibodies but has not previously been reported in anti-NXP-2 DM.^8^, ^9^, ^10^ Pseudoangioedema of the lips is a rare cutaneous manifestation of DM, and this unusual feature may serve as a clue to promote prompt diagnosis.

## Conflicts of interest

None disclosed.
